# Cadmium Impairs Albumin Reabsorption by Down-regulating Megalin and ClC5 Channels in Renal Proximal Tubule Cells

**DOI:** 10.1289/ehp.0901874

**Published:** 2010-06-24

**Authors:** Patrizia Gena, Giuseppe Calamita, William B. Guggino

**Affiliations:** 1 Department of Physiology, Johns Hopkins University School of Medicine, Baltimore, Maryland, USA; 2 Department of General and Environmental Physiology, University of Bari, Bari, Italy

**Keywords:** albuminuria, cadmium, ClC5, heavy metals, megalin, nephrotoxicity, renal proximal tubules

## Abstract

**Background:**

Cadmium (Cd) is a potent nephrotoxicant that impairs the reabsorptive and secretory functions of the renal proximal tubule, leading to albuminuria.

**Objectives:**

To gain insights into the mechanisms of Cd-induced albuminuria, we investigated effects of Cd on the expression of megalin and chloride channel 5 (ClC5), two key players in albumin- receptor–mediated endocytosis.

**Methods:**

We used quantitative polymerase chain reaction, Western blotting, the albumin endocytosis assay, and confocal microscopy to evaluate effects of Cd on the expression and regulation of megalin and ClC5 in cultured LLC-PK1 cells, a pig proximal tubular cell model.

**Results:**

Ten micromolar cadmium chloride (CdCl_2_) caused a significant time- and dose-dependent decrease in both mRNA and protein levels of megalin and ClC5, whereas no changes resulted from exposure to other divalent metals (zinc chloride, manganese chloride, magnesium chloride, and nickel chloride). After inhibiting protein synthesis using cycloheximide (CHX), we found that levels of both megalin and ClC5 were lower in Cd-challenged cells than in cells treated with Cd or CHX only, which is consistent with reduced translation and/or posttranslational down-regulation. Moreover, Cd-induced degradation of megalin and ClC5 was abolished by the lysosomal pathway inhibitor bafilomycin A-1 but not by the proteasome system blocker MG-132, suggesting that the enhanced proteolysis was occurring via lysosomes. Using confocal microscopy, we observed a remarkable reduction of fluoroisothiocyanate (FITC)-labeled albumin uptake after Cd exposure.

**Conclusions:**

We found that Cd reduced the transcriptional expression of megalin and *ClC5* and, at the same time, increased the degradation of megalin and ClC5 proteins via the lysosomal pathway in an *in vitro* model of renal proximal tubular cells. Overall, these results provide valuable insights into the molecular mechanisms by which Cd impairs luminal protein reabsorption by renal proximal tubules.

Cadmium (Cd) is a well-known occupational and environmental hazard with a potent nephrotoxic action. Cd concentrates in several vital organs, among which liver and kidney are the primary targets that accumulate and are affected adversely by this toxic heavy metal (Zalups and Ahmad 2002). A body of studies provided meaningful insights into the Cd uptake and accumulation into renal cells (Endo and Shaikh 1993; Zalups and Ahmad 2002). Once within the cell, Cd reacts with thiol groups and may substitute for zinc (Zn) in critical metabolic processes, but it can also cause DNA single-strand breaks, lipid peroxidation, and generation of oxidatively damaged proteins (Bertin and Averbeck 2006). Enterally absorbed Cd is taken up by the liver, where a significant amount of its ionized form (Cd^2+^) is bound to metallothioneins (MTs), leading to hepatocellular necrosis and/or apoptosis, with the consequent release of Cd–MT complexes into the bloodstream. Some of these complexes are delivered to the kidneys, where they are filtered by the glomeruli and reabsorbed by the proximal tubules (Zalups and Ahmad 2002). Kidneys can accumulate up to 50% of the total body burden of Cd in subjects occupationally and environmentally exposed (Järup 2002), causing a decrease in tubular reabsorption and leading to proximal tubulopathies characterizing renal Fanconi’s syndrome (Hamada et al. 1997; Prozialeck et al. 1993). Although Cd-induced nephrotoxicity is widely studied, the molecular mechanisms underlying the damage and subsequent regeneration of the renal tubular epithelium remain elusive.

An extensive endocytic apparatus, located in the apical membrane of proximal tubule cells, plays a key role in the reabsorption and degradation of glomerular-filtered albumin and low-molecular-weight proteins (Marshansky et al. 2002) and in the recycling of many functionally important apical membrane transporters (Brown and Stow 1996). Human kidney is able to reabsorb 95% of all filtered albumin via receptor-mediated endocytosis within proximal tubules (Nielsen 1993). Albumin endocytosis requires a macromolecular protein complex formed by the megalin/cubilin scavenger receptor Na^+^-H^+^ exchanger isoform 3 (NHE3), vacuolar proton-ATPase (v-H^+^-ATPase), and chloride channel 5 (ClC5). The megalin/cubilin receptor binds albumin, which is subsequently internalized by clathrin-coated pits into endosomes that are acidified by NHE3 and v-H^+^-ATPase. The anion shunt is provided by ClC5 (Christensen and Birn 2001; Hryciw et al. 2006).

Previous reports have shown *in vivo* that Cd alters the expression of NHE3 and v-H^+^-ATPase (Ahn et al. 2005; Herak-Kramberger et al. 1998). To take this one step further, we examined whether Cd exposure affects the expression of the two other proteins involved in renal albumin endocytosis: megalin and ClC5. In proximal tubule cells, megalin, a 600-kDa transmembrane glycoprotein, interacts synergistically with cubulin as scavenger for the luminal uptake of a large number of proteins, including albumin (Verroust et al. 2002). Moreover, ClC5 is mainly expressed in the early endosomes of the renal proximal tubule and intercalated cells of the cortical collecting duct (Jentsch et al. 2002; Piwon et al. 2000). Heterologous expression of ClC5 in *Xenopus laevis* oocytes or HEK 293 cells (Devuyst et al. 1999) showed that it mediates plasma membrane currents (Schriever et al. 1999). Because ClC5 acts as a chloride/proton exchanger when activated by positive voltages (Picollo and Pusch 2005), it is believed to play a critical role in the endosomal acidification as an antiporter by coupling chloride ion (Cl^−^) gradients to vesicular pH gradients (Picollo and Pusch 2005). Knockout mice lacking functional megalin or ClC5 have both albuminuria and low-molecular-weight proteinuria, hallmarks of renal Fanconi’s syndrome (Christensen et al. 2003; Leheste et al. 1999; Piwon et al. 2000; Wang et al. 2000). This led us to investigate effects of Cd on the expression, subcellular distribution, and possible functional implications of both megalin and ClC5 in the Cd-induced dysregulation of renal proximal tubule albumin reabsorption.

## Materials and Methods

### Cell culture

We obtained a pig renal proximal tubule cell line (LLC-PK1) from American Type Culture Collection (ATCC, Rockville, MD, USA). LLC-PK1 cells were grown in low-glucose Dulbecco’s modified Eagle’s medium (DMEM) supplemented with 10% fetal bovine serum, 50 U/mL penicillin, and 10 ng/mL streptomycin (Invitrogen, Carlsbad, CA, USA) in a humidified atmosphere of 5% CO_2_/95% air at 37°C. Cells were grown and subcultured every week on Transwell inserts (pore size, 0.4 μm; Fisher, Newark, DE, USA). Cell monolayers were used 1 day postconfluence, typically 7 days after seeding.

### Treatment with Cd and divalent metals manganese (Mn), magnesium (Mg), zinc (Zn), and nickel (Ni)

Cd, Zn, Mn, Mg, and Ni chloride salt forms were purchased from Sigma Chemicals (St. Louis, MO, USA). Confluent cell monolayers were washed twice with phosphate-buffered saline (PBS) and incubated with serum-free medium containing the suitable concentration of all divalent metals for 9 hr at 37°C. Divalent metals were added both to the lower (10 μM) and to the upper (1 μM) compartment of the Transwell clusters corresponding to the basolateral and the apical membrane side of the cells, respectively. In the time course experiments, cells were treated with 10 μM CdCl_2_ for 3–24 hr. After the exposure, Cd-treated cells were washed three times with PBS to remove the residual metals. Untreated cells were incubated only with the serum-free DMEM and treated as the cells exposed to the metals.

### CHX, bafilomycin A1, and MG-132 treatments

Cycloheximide (CHX), bafilomycin A1 (Sigma), and carbobenzoxy-l-leucyl-l-leucyl-l-leucinal (MG-132) (Calbiochem, San Diego, CA, USA) were dissolved in dimethyl sulfoxide (DMSO). LLC-PK1 cells were incubated with serum-free medium containing each chemical or DMSO alone at the same concentration used in treated cells (0.1% or 0.05%).

### Cytotoxicity studies

Cd-induced cytotoxicity and cellular damage were both evaluated by trypan blue exclusion, 3-(4,5-dimethylthiazol-2-yl)-2,5-diphenyltetrazolium-bromide (MTT), and lactate dehydrogenase leakage (LDH) assays. LLC-PK1 cells were plated at 1 × 10^6^ cells/dish and cultured for 2–3 days in a serum-free medium. The trypan blue exclusion test was carried out according to the manufacturer’s (Sigma) instructions. For the MTT assay, both control and Cd-treated cells were incubated for 4 hr with 0.8 mg/mL MTT dissolved in serum-free medium. Washings with PBS were followed by adding DMSO and shaking for 10 min to achieve the complete dissolution. Aliquots of the resulting solutions were transferred into 96-well plates, and absorbance was recorded spectrophotometrically at 570 nm. The LDH assay, a colorimetric test to evaluate cell integrity, was performed using a cytotoxicity detection kit from Roche Diagnostics GmbH (Mannheim, Germany).

### Quantitative real-time reverse-transcriptase polymerase chain reaction (qRT-PCR)

We extracted total RNA from LLC-PK1 cells using the RNeasy Mini Kit (Qiagen, Valencia, CA, USA). One microgram of total RNA from each sample was reverse transcribed to cDNA. Equal amounts of the resulting cDNA were used as templates for the subsequent quantitative real-time RT-PCR reactions that were set up using the following final concentrations: 200 nM each of forward and reverse primers [see Supplemental Material, Table 1 (doi:10.1289/ehp.0901874)], 1× SYBR Green PCR Master Mix (Applied Biosystems, Foster City, CA, USA), and 1.0 μL cDNA. Quantitative real-time RT-PCR was carried out using the following cycling conditions: initial incubation (50°C, 2 min), Taq activation (95°C, 3 min), and 40 cycles of denaturation (95°C, 1 min), annealing (60°C, 1 min), and extension (72°C, 1 min). After 40 PCR cycles, the temperature was increased from 72°C to 95°C to construct a melting curve. Data were normalized against β-actin.

### Western blotting

After the treatments, cells were washed twice with PBS and harvested with lysis buffer [60 mM HEPES, (pH 7.5), 150 mM NaCl, 3 mM KCl, 5 mM EDTA, 5 mM EGTA, 1% Triton X-100, and complete protease inhibitor cocktail (all from Roche Diagnostics, Indianapolis, IN, USA)]. Lysates were insulin-syringe homogenized and centrifuged at 6,000 × *g* for 10 min at 4°C. The resulting supernatant was retained, and the protein concentration was determined using the BCA Protein Assay Kit (Pierce, Rockford, IL, USA). Equal protein amounts (40 μg) were incubated in Laemmli buffer for 30 min at 37°C and electrophoresed in 7.5% SDS/PAGE gels (Bio-Rad, Hercules, CA, USA). Resolved proteins were then transferred onto a polyvinyl difluoride membrane and blocked in 5% (wt/vol) low-fat milk in blocking buffer (20 mM Tris-HCl, 0.15 mM NaCl, 1% Triton X-100, pH 7.5) for 1 hr at room temperature. Blots were incubated overnight with sheep anti-megalin (kindly provided by O. Devuyst, Louvain, Belgium), goat polyclonal anti-ClC5 (ClC5-D17), or anti-actin (I-19) antibodies (Santa Cruz Biotechnology, Santa Cruz, CA, USA). Proteins were detected with SuperSignal West Dura Extended Duration Substrate (Pierce). Acquired images were analyzed by Image J software (Rasband 2009).

### Albumin endocytosis assay and immunofluorescent staining

The albumin endocytosis assay was performed as previously described (Wang et al. 2005). Briefly, LLC-PK1 cells were grown on Transwell inserts in supplemented DMEM for 7 days to ensure that the cells were polarized before the experiments. After Cd exposure for 9 hr, cells were washed twice with PBS supplemented with 1.8 mM CaCl_2_ and 1.0 mM MgCl_2_ (PBS^2+^) and exposed from their apical surfaces to prewarmed fluoroisothiocyanate (FITC)-labeled albumin (0.5 mg/mL) at 37°C for 15 min. Cells held at 4°C to abolish the endocytic process were used as negative controls. Before fixation, cells were put on ice and washed with ice-cold PBS^2+^ eight times to block albumin endocytosis. Then, they were fixed with 4% paraformaldehyde at room temperature for 15 min, permeabilized with 0.1% Triton X-100 for 1–2 min, and blocked with 0.1% gelatin in PBS^2+^ for 30 min. Cell monolayers were incubated with sheep anti-megalin or rabbit anti-ClC5 antibodies (Millipore, Temecula, CA, USA) in blocking solution for 1 hr, washed with PBS^2+^ three times, and incubated with the appropriate secondary antibody conjugated to Cy3 fluorescent dye (Jackson Immunoresearch, West Grove, PA, USA). In some experiments, cells were fixed and immunostained with fluorescent wheat germ agglutinin (WGA; Invitrogen) for 1 hr. After staining, the Transwells were washed thoroughly with PBS and mounted with an antiquenching medium. Finally, slides were sealed and viewed with a Zeiss Axiovert 200 fluorescence microscope equipped with 510-Meta confocal module (Carl Zeiss, Oberkocken, Germany) and fitted with a 63× oil-immersion objective lens. Fluorescent images were acquired and analyzed by measuring the average intensity per cell from multiple images after background subtraction using IPLAB software (BD Biosciences, San Jose, CA, USA).

### Statistical analysis

We used Student’s *t*-test to determine the statistical significance. Results were expressed as mean ± SE and calculated based on three to five independent experiments. A *p*-value < 0.05 was considered to be statistically significant.

## Results

### Cd down-regulates gene expression of megalin and ClC5 in proximal tubule cells

**Figure 1:**
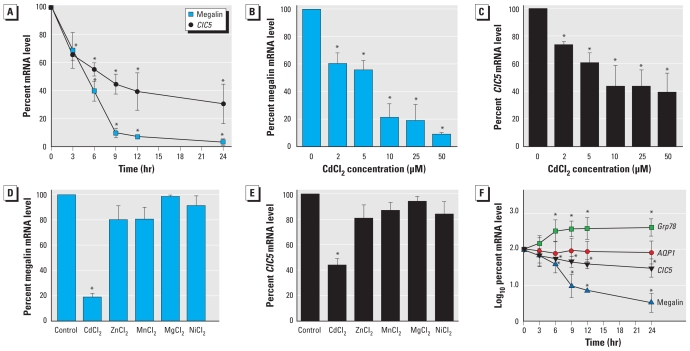
Cd reduces the gene expression of megalin and *ClC5* in LLC-PK1 cells compared with controls. (*A*) Time course of megalin and *ClC5* gene expression in LLC-PK1 cells incubated with 10 μM CdCl_2_ for 3–24 hr, showing large decreases in *ClC5* and megalin mRNA levels in response to Cd. (*B* and *C*) Dose-dependent effects of Cd on megalin (*B*) and *ClC5* (*C*) mRNA levels upon treatment of proximal tubule cells with 0, 2, 5, 10, 25, or 50 μM CdCl_2_ for 9 hr. (*D* and *E*) Quantitative real-time RT-PCR histograms showing megalin (*D*) and *ClC5* (*E*) mRNA levels in LLC-PK1 cells incubated with 10 μM of each divalent metal compound for 9 hr. Unlike the Cd challenge, cells exposed to various divalent metals showed no effects on megalin and *ClC5* mRNA levels; the purity of all divalent metals was ≤ 5 ppm of heavy metals contamination. (*F*) Quantitative real-time RT-PCR performed on LLC-PK1 cells treated with 10 μM CdCl_2_ for 3–24 hr; using specific primers for *Grp78*, *Aqp1*, megalin, *ClC5*, and β-actin indicates that the inhibitory effects of Cd are specific to megalin and *ClC5* gene expression. All values were normalized against β-actin expression; control values (time zero for *A* and *F*, and absence of CdCl_2_ for *B–E*) were set to 100%. Values are mean ± SE of four experiments. *p < 0.05 compared with untreated controls.

We performed quantitative real-time RT-PCR to evaluate time course and dose effects of CdCl_2_ treatment on megalin and *ClC5* mRNA expression in proximal tubule cells. In LLC-PK1 cells incubated with 10 μM CdCl_2_, the mRNA levels of both megalin and *ClC5* decreased significantly until 9 hr of treatment (−90.3 ± 3.3% and −55.4 ± 7.1%, respectively) and remained consistently low at 24 hr (Figure 1A). When cells were treated with 2–50 μM CdCl_2_ for 9 hr, both megalin and *ClC5* mRNA levels were reduced in a dose-dependent manner (Figure 1B,C). Interestingly, megalin expression was much more sensitive to Cd than was *ClC5* expression. Megalin and *ClC5* mRNA expressions were normalized against β-actin expression, which was unaffected by CdCl_2_ exposure (Figure 1A–C). At 9 hr of Cd treatment, cell viability (assayed by trypan blue exclusion) was unchanged at concentrations ≤ 10 μM but decreased by 38% and 45% at 25 and 50 μM CdCl_2_, respectively (data not shown). We confirmed absence of intoxication with exposures up to 25 μM CdCl_2_ using MTT and LDH assays [see Supplemental Material, Figure 1 (doi:10.1289/ehp.0901874)]. Metabolic activity and cellular integrity seen in our experimental conditions were thoroughly in line with previous studies that used confocal microscopy, flow cytometry, and LDH cytotoxicity assays to show that Cd induces apoptosis in LLC-PK1 cells only at relatively high Cd concentrations (50–100 μM) and after a prolonged exposure (24 hr) (Alvarez-Barrientos et al. 2001; Gennari et al. 2003). Because CdCl_2_ in aqueous solution is known to cause acute nephrotoxicity in cultured proximal tubule cells (Barbier et al. 2005; Liu et al. 1994), we carried out a Cd challenge using *in vitro* experiments in which we exposed cultured LLC-PK1 cells to low micromolar concentrations of Cd^2+^. All of the experiments except the dose responses (Figure 1B,C) used 10 μM CdCl_2_.

### CdCl_2_ specifically reduces megalin and ClC5 transcript levels

To determine whether the decrease in megalin and *ClC5* at the gene level after Cd treatment was specific to this metal, we incubated LLC-PK1 cells for 9 hr with 10 μM of other divalent metals such as Zn^2+^, Mn^2+^, Mg^2+^, and Ni^2+^. Cell viability, measured by trypan blue exclusion assay, was 92.8 ± 3.7% for CdCl_2_, 97.5 ± 2.4% for ZnCl_2_, 96.3 ± 1.9% for MnCl_2_, 98.8 ± 0.4% for MgCl_2_, and 93.3 ± 6.6% for NiCl_2_. Quantitative real-time RT-PCR results showed that, among the metals examined, only Cd treatment significantly reduced the mRNA levels of megalin and *ClC5* in LLC-PK1 cells (Figure 1D,E), indicating that the Cd-induced down-regulation of megalin and *ClC5* gene expression was not a general effect exerted by divalent metals. However, the most relevant control was Zn, which, like Cd, is another group IIB transition metal. To evaluate whether CdCl_2_ caused a general down-regulation of gene expression in proximal tubule cells, we compared the transcript levels of megalin and *ClC5* with those of 78-kDa glucose-regulated protein (*Grp78*), a well-known Cd-inducible target (Liu et al. 2006), and aquaporin-1 (*Aqp1*), a plasma membrane protein unaffected by CdCl_2_ treatment (Sabolic et al. 2002). As shown in Figure 1F, CdCl_2_ exposure resulted in a time-dependent increase in *Grp78* mRNA expression, whereas it had no effect on *Aqp1* mRNA levels. These results suggested that the inhibitory effects of Cd on megalin and *ClC5* mRNA in LLC-PK1 cells were specific to CdCl_2_ and could not be attributed to an overall down-regulation of gene expression resulting from cytotoxicity.

### Time course of CdCl_2_-induced down- regulation of megalin and ClC5 protein expression

**Figure 2:**
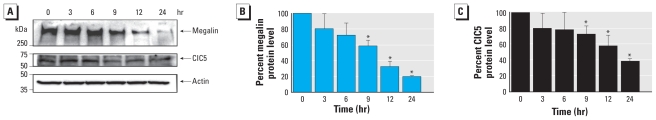
Megalin and ClC5 protein levels are remarkably decreased in Cd-treated proximal tubule cells compared with controls. (*A*) Representative Western blots of cell lysates (prepared from LLC-PK1 cells exposed to 10 μM CdCl_2_ for 3–24 hr) incubated with megalin, ClC5, and actin antibodies; Cd exposure significantly reduced megalin and ClC5 protein levels starting at 9 hr, with a peak reduction at 24 hr. (*B* and *C*) Densitometric analyses of megalin (*B*) and ClC5 (*C*) protein from Western blots shown in *A*. All values were normalized against β-actin expression; the control value (time zero) was set to 100%. Values are mean ± SE of four experiments. *p < 0.05 compared with untreated controls.

We performed immunoblotting experiments to study time- and dose-dependent effects of Cd exposure on both megalin and ClC5 protein levels. Consistent with the results of quantitative real-time RT-PCR, in LLC-PK1 cells treated with 10 μM CdCl_2_, megalin and ClC5 protein expression began to decrease significantly after 9 hr (−41.2 ± 7.2% and −27.7 ± 10.2%, respectively), with a peak reduction at 24 hr (−80.2 ± 1.4% and −61.6 ± 3.1%, respectively) (Figure 2A–C). In agreement with the mRNA studies, megalin appeared to be more sensitive to Cd than was ClC5. The protein levels of megalin and ClC5 were normalized against β-actin, which was unaltered after 2–24 hr of CdCl_2_ exposure (Figure 2A–C). Experiments to evaluate megalin and ClC5 protein expressions after exposure to increasing doses of CdCl_2_ for 9 hr also supported dose-dependent effects, with stronger effects on megalin than on ClC5 [see Supplemental Material, Figure 2 (doi:10.1289/ehp.0901874)].

### Cd-induced down-regulation of megalin and ClC5 proteins is due to an increase of their lysosomal degradation

**Figure 3:**
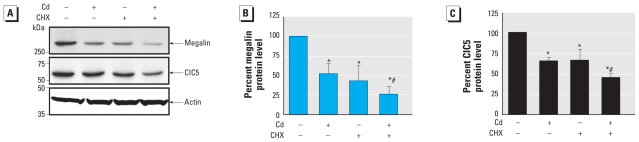
Megalin and ClC5 are reduced in presence of Cd and CHX, a protein synthesis inhibitor, compared with controls. (*A*) Representative Western blots of cell lysates [prepared from LLC-PK1 cells incubated for 9 hr with 10 μM CdCl_2_, 10 μg/mL CHX, 10 μg/mL CHX plus 10 μM CdCl_2_ or serum-free medium containing 0.1% DMSO (the same concentration used in treated cells)] incubated with megalin, ClC5, and actin antibodies. Cd treatment plus CHX significantly decreased levels of both proteins, especially megalin, relative to levels in cells treated with Cd or CHX only. (*B* and *C*) Densitometric analyses of megalin (*B*) and ClC5 (*C*) protein from Western blots shown in *A*. Values were normalized against β-actin expression, and the control value (without CdCl_2_ and/or CHX) was set to 100%. Values are mean ± SE of three experiments. *p < 0.05 compared with untreated controls. ^#^*p* < 0.05 compared with cells treated with Cd only.

**Figure 4:**
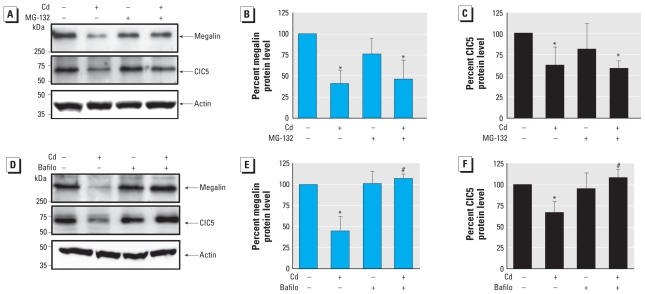
Megalin and ClC5 undergo enhanced degradation via the lysosomal pathway. (*A*) Representative Western blots of cell lysates from LLC-PK1 cells incubated for 9 hr with 10 μM CdCl_2_, 10 μM MG-132, or 10 μM MG-132 plus 10 μM CdCl_2_. (*B* and *C*) Densitometric analyses of megalin (*B*) and ClC5 (*C*) protein from Western blots shown in *A*. (*D*) Western blots of cell lysates from LLC-PK1 cells exposed for 9 hr to 10 μM CdCl_2_, 1 μM bafilomycin A-1 (Bafilo), or 1 μM Bafilo plus 10 μM CdCl_2_. (*E* and *F*) Densitometric analyses of megalin (*E*) and ClC5 (*F*) protein from Western blots shown in *D*. Untreated controls were incubated with serum-free medium containing 0.1% DMSO, the same concentration used in treated cells. For *B, C, D,* and *F*, all values were normalized against β-actin expression, and the control values were set to 100%. Values are mean ± SE of three experiments. *p < 0.05 compared with untreated controls. ^#^*p* < 0.05 compared with cells treated with Cd only.

To address the possible molecular mechanism by which Cd dysregulated megalin and ClC5 both at the protein and at the gene levels, we incubated LLC-PK1 cells with 10 μg/mL of CHX, a protein synthesis inhibitor, in the presence or absence of 10 μM CdCl_2_ for 9 hr. CHX treatment significantly reduced the expression of both proteins in CdCl_2_-challenged cells (−74.4 ± 10.4% for megalin, and −50.3 ± 11.2% for ClC5) compared with untreated cells and cells treated with CdCl_2_ or CHX only (Figure 3A–C). Results showing reduced abundance of megalin and ClC5 protein upon treatment with CdCl_2_ and CHX are consistent with reduced protein expression due to effects on transcription but might also reflect a Cd-induced increase in proteolytic degradation of both proteins via lysosomal or proteasomal pathways. To assess whether the reduction in both megalin and ClC5 protein expression was due to an effect of Cd on protein degradation dependent on the ubiquitin/proteasome or the lysosomal pathways, we treated LLC-PK1 cells with 10 μM MG-132, a proteasome inhibitor (Rock et al. 1994), or 1 μM bafilomycin A1, a lysosomal proton pump inhibitor (Yoshimori et al. 1991), in the presence or absence of 10 μM CdCl_2_ for 9 hr. Megalin and ClC5 proteins were still significantly reduced in CdCl_2_-treated cells exposed to MG-132 (Figure 4A–C). In contrast, when cells were treated with bafilomycin A1, the effects of Cd on megalin and ClC5 protein levels were completely abolished, with levels remaining unchanged (Figure 4D–F). We observed no significant change in protein abundance in LLC-PK1 cells treated with bafilomycin only, consistent with an effect of Cd on lysosomal protein degradation. Overall, these results indicated that, in addition to reducing mRNA levels (Figure 1), Cd promoted the degradation of megalin and ClC5 proteins via lysosomes rather than via proteasomes.

### Effect of CdCl_2_ on albumin endocytosis

**Figure 5:**
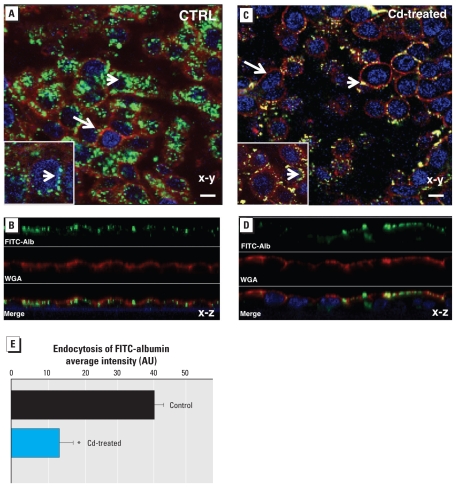
FITC-albumin endocytosis is impaired in Cd-challenged proximal tubule cells. (*A* and *B*) FITC-albumin endocytosis assay using LLC-PK1 cells incubated in control conditions (without CdCl_2_; CTRL) and exposed for 15 min to 0.5 mg/mL FITC-labeled albumin. Albumin internalization is shown by the numerous fluorescent vesicles (green) located in the subapical (arrows) and intracellular (arrowheads) compartments. (*C* and *D*) Confocal imaging of FITC-albumin endocytosis by LLC-PK1 cells after 9 hr treatment with 10 μM CdCl_2_ and 15 min exposure to 0.5 mg/mL FITC-labeled albumin; FITC-albumin uptake was remarkably reduced in CdCl_2_-treated cells compared with the control cells. Insets: colocalization (arrowhead; yellow) between rhodamine-labeled WGA (red), an apical membrane marker, and FITC-albumin (green) in CdCl_2_-exposed cells (*B*) compared with control cells (*A*). Nuclei are labeled with 4′,6-diamidino-2-phenylindole (DAPI; blue). In *A* and *C*, bars = 20 μm; for *B* and *D,* magnification, 63×. For insets in *A* and *B*, magnification, 100×. (*E*) Quantitative analysis of FITC-albumin endocytosis, measured as average fluorescence intensity per cell, in control and Cd-challenged cells. Values are mean ± SE of five experiments. *p < 0.05 compared with untreated controls.

To investigate the effect of CdCl_2_ on albumin endocytosis, we preincubated LLC-PK1 cell monolayers with 10 μM CdCl_2_ for 9 hr and then treated them with 0.5 mg/mL FITC-labeled albumin for 30 min at 37°C. Consistent with previous reports (Choi et al. 1999; Lebeau et al. 2001), confocal microscopy showed that FITC-albumin uptake was remarkably reduced in Cd-treated cells (Figure 5C,D) compared with controls (Figure 5A,B). By quantitative analysis, the FITC-albumin uptake of Cd-treated monolayers, measured as average fluorescence intensity per cell, was decreased by 68 ± 10% compared with untreated control cells (Figure 5E). In line with the observed dose dependence of the Cd effect, we observed no changes in FITC-albumin uptake with lower doses (2–5 μM) of Cd^2+^ (data not shown). No FITC-albumin uptake was seen in LLC-PK1 cells exposed to FITC-albumin at 4°C (negative controls). In contrast with the untreated control cells, which showed an accumulation of FITC-albumin in the intracellular compartment (Figure 5A, green), in Cd-challenged cells FITC-albumin colocalized (yellow; Figure 5B) with rhodamine-labeled WGA (red), a well-known apical membrane marker (Sambuy et al. 1988). These observations suggest that CdCl_2_ exposure blocked apical receptor-mediated FITC-albumin endocytosis and thereby prevented internalization of albumin into the cytoplasmic compartment of the proximal tubule cells.

Confocal microscopy of labeled FITC-albumin and megalin or ClC5 in CdCl_2_-treated cells indicated a simultaneous decrease in FITC-albumin and both proteins [see Supplemental Material, Figure 3 (doi:10.1289/ehp.0901874)].

## Discussion

Kidney proximal tubule cells are among the major targets of Cd-induced renal injury (Van Vleet and Schnellmann 2003). However, the subcellular mechanism underlying Cd nephrotoxicity remains poorly understood. We carried out this study based on the working hypothesis that the enhanced albuminuria that characterizes Cd-treated proximal tubule cells is due to impaired endocytosis caused by dysregulatory effects exerted by Cd^2+^ on megalin and ClC5, two proteins that play key roles in albumin-receptor–mediated endocytosis. *In vivo*, Cd-MT treatment exerted acute effects on both the abundance and subcellular distribution of megalin (Sabolic et al. 2002). In our study we took these results a step further to provide a deeper understanding of the mechanisms and molecular pathways involved in Cd nephrotoxicity using a well-established *in vitro* renal cell model. Our results show down-regulation of both megalin and ClC5 at both the protein and gene levels in a time- and dose-dependent manner. We observed no change in the transcriptional expression of megalin and ClC5 in LLC-PK1 cells treated with other divalent metals (Zn^2+^, Mn^2+^, Mg^2+^, and Ni^2+^), indicating that this effect was specific to Cd^2+^. In addition, effects of Cd^2+^ on megalin and ClC5 were not the consequence of mere cellular damage. After Cd exposure, altered expression of megalin and ClC5 may reflect the distinct accumulation and biochemical properties of this toxic metal. Because Cd is not a redox-active metal, it is reasonable to assume that it is not directly involved in Fenton-type reactions and does not generate free radicals by itself (Stohs and Bagchi 1995). However, Cd can induce oxidative stress indirectly by reducing cellular antioxidants and causing the release of reactive oxygen species (ROS) by mitochondria (Waisberg et al. 2003). Hence, based on previous reports (Thévenod and Friedmann 1999), we hypothesize that Cd-induced reduction of megalin and *ClC5* mRNA levels is the consequence of an indirect effect of this heavy metal on DNA transcription involving ROS production and oxidative stress. Our observation that cells treated with Cd and CHX (to inhibit protein synthesis) had reduced protein abundance relative to cells treated with Cd or CHX alone may be partly explained by reduced transcription, but might also reflect increased degradation of megalin and ClC5 in Cd-challenged proximal tubule cells. The fact that protein levels in cells treated with Cd and bafilomycin A-1, a lysosomal pathway inhibitor, were comparable to protein levels in untreated cells suggests that Cd^2+^ targets megalin and ClC5 for enhanced proteolysis through lysosomes. In contrast, protein levels were comparable in Cd-treated cells with or without MG-132, a proteasome system blocker. In kidney proximal tubule cells, Cd-mediated oxidative stress leads to degradation of Na^+^/K^+^-ATPase through the proteasomal and endo-/lysosomal pathways (Thévenod and Friedmann 1999). Similarly, it is reasonable to speculate that Cd acts on megalin and ClC5 expression simultaneously and at two distinct levels: by reducing transcription of megalin and *ClC5* mRNA, and by increasing megalin and ClC5 degradation through the lysosomal pathway. It is therefore conceivable that the changes in mRNA levels observed in response to Cd^2+^ did not necessarily determine the level of megalin and ClC5 protein expression. The remarkable inhibition of albumin uptake and internalization, which was associated with a significant reduction in the expression of megalin (at the cell margins and in intracellular vesicles) and ClC5 (in intracellular vesicles) in Cd-challenged cells, was consistent with the recent work by Lebeau et al. (2001), who reported that exposing opossum kidney cell monolayers to Cd for 24 hr inhibited the endocytic mechanism of protein reabsorption and that such inhibition was due to a Cd-induced reduction in the number of recycling receptors that internalize low-molecular-weight proteins. However, the colocalization of WGA and albumin that we observed indicated that albumin was bound to its plasma membrane receptor in LLC-PK1 cells even after Cd treatment. This suggests either that megalin is not primarily important for binding albumin, so that other receptors can substitute for megalin, or that reduced megalin expression is not sufficient to abolish albumin binding completely. The same effect of Cd on megalin and ClC5 expression was previously observed for other proteins involved in albumin-receptor–mediated endocytosis, including NHE3 and v-H^+^-ATPase (Ahn et al. 2005; Herak-Kramberger et al. 1998).

Overall, our data suggest that the nephrotoxicant action of Cd causes a global down-regulation of all proteins involved in the macromolecular complex required for albumin-receptor–mediated endocytosis, with consequent albuminuria. This may be of clinical and diagnostic significance because albuminuria per se is well recognized as a cause of tubular damage and as a biomarker of renal and cardiovascular diseases. Therefore, improving knowledge of the molecular mechanisms of Cd toxicity may reveal information of therapeutic relevance for renal diseases with associated albuminuria.
